# A Heart Gone Bananas: Allergy-Induced Coronary Vasospasm due to Banana (Kounis Syndrome)

**DOI:** 10.1155/2023/5987123

**Published:** 2023-06-23

**Authors:** Lauren Reinhold, Stephen Lynch, Carl B. Lauter, Simon R. Dixon, Andrew Aneese

**Affiliations:** ^1^William Beaumont University Hospital, Royal Oak, MI, USA; ^2^Oakland University William Beaumont School of Medicine, Rochester, MI, USA

## Abstract

Kounis syndrome encompasses a variety of cardiovascular signs and symptoms associated with mast cell activation in the setting of allergic or hypersensitivity and anaphylactic or anaphylactoid insults. It can manifest as coronary vasospasm, coronary, or in-stent thrombosis, and acute myocardial infarction with plaque rupture. Various medications as well as foods including fish, shellfish, mushroom, kiwi, and rice pudding have been implicated as causal agents. We present what we believe to be the first documented case of Kounis syndrome manifesting as coronary vasospasm as the result of an allergy to banana. This case highlights the importance of considering allergic causes of angina and allergy referral in a patient with known atopy and an otherwise negative cardiovascular workup. It also emphasizes to consider food allergy, especially banana, as a cause of Kounis syndrome.

## 1. Introduction

Kounis syndrome is a rare cause of acute coronary syndrome due to allergy [1]. It was first described in 1981 after intravenous histamine was injected into patients causing coronary vasospasm [2]. It has now been divided into three variants. Multiple medications and foods have been shown to induce Kounis syndrome. We report the first case of Type I Kounis syndrome due to an allergy to banana.

## 2. Case Report

A 56-year-old woman initially presented with daily, intermittent, substernal angina exacerbated by exertion and cool temperatures. Her angina was only relieved with sublingual nitroglycerin, which she used up to 10 times daily. She has a past medical history of asthma, severe contrast allergy and allergies to multiple medications, and a remote history of Hodgkin's. Her family history is significant for atopy, asthma, and coronary artery disease. Initial cardiac workups included negative troponin levels and normal electrocardiograms. A stress test showed anterolateral ECG changes but no perfusion defects on imaging. Coronary catheterization revealed normal coronaries. She was diagnosed as having variant angina with vasospasm, and was started on a trial of calcium-channel blockers without symptomatic relief. Given her history of atopy, she was referred to our allergy clinic about 1 year after initial work up began. Tryptase levels were checked which were found to be persistently elevated, 13.3 and 13.6 ng/mL. Allergy specific IgE testing for banana, latex, and birch were checked after the patient reported perioral itching and tingling with banana ingestion. Her IgE testing resulted with positivity for banana 1.31 kU/L, birch 3.02 kU/L, and latex 0.43 kU/L. She had been eating multiple bananas daily as part of what she believed to be a “heart-healthy” diet. Upon discontinuing bananas from her diet, her chest pain improved dramatically both in severity and frequency. Multiple tryptase levels taken after the discontinuation of bananas have been within normal range, 8.4 and 8.1 ng/mL, and she has remained free of chest pain.

## 3. Discussion

Kounis syndrome is the occurrence of an acute coronary syndrome that arises in conjunction with hypersensitivity, allergic, anaphylactic, or anaphylactoid reactions [1, 3]. The association of histamine release in allergic reactions and coronary vasospasm was first described in 1981 after injection of intravenous histamine in patients with nonobstructive coronary artery disease produced coronary artery spasm in 4 out of 12 patients [2]. In 2016, Kounis defined three different variants [1, 3]. Type I Kounis syndrome occurs in patients without underlying coronary artery disease and manifests as chest pain from coronary vasospasm. Type II occurs in patients with underlying coronary artery disease and manifests as an acute myocardial infarction. Type III manifests as in-stent thrombosis and can be further divided into Type IIIa: stent thrombosis due to allergy and Type IIIb: stent restenosis due to allergy. These variants of Kounis syndrome occur in 72.6%, 22.3%, and 5.1% of patients, respectively [3]. Despite the clinical implications surrounding this entity, Kounis syndrome is likely underrecognized and underdiagnosed.

The pathophysiology behind this syndrome predominately involves mast cells and their interaction with other immune cells [1]. During an allergic reaction, mast cell degranulation and inflammatory mediator release occurs. It has been speculated that the heart and coronary arteries might be the primary target of allergic or anaphylactic reactions [4]. The effects of these mediators may lead to serious cardiac events including chest pain, ECG changes, arrhythmias, and even myocardial ischemia and infarction. Various triggers of Kounis syndrome have been described in recent years. The most common causes include medications, mainly antibiotics or anti-inflammatory drugs. Environmental antigens and insect bites have also been implicated [1, 3]. Although uncommon, food-related triggers have also been described. These food triggers include ingestion of certain raw or undercooked fish, scombroid poisoning from spoiled fish, shellfish, gelofusin substance, mushroom, rice milk, kiwifruit, tomato salad, and certain nuts [1, 3]. To our knowledge, Kounis syndrome related to banana ingestion has not been previously reported.

In the general population, banana fruit is an uncommon cause of allergy, affecting around 0.6% of people [5]. In atopic individuals, it has been shown to cause allergy more commonly. Banana fruit allergy results from an abnormal immune response to banana proteins with six major proteins identified: Mus a1 (proflin-actin binding protein), Mus a 2 (Class 1 chitinase), Mus a 3 (nonspecifc lipid transfer protein), Mus a 4 (thaumatin-like protein), Mus a 5 (beta 1,3 glucanase), and Mus a 6 (ascorbate peroxidase) [5]. Multiple different proteins have been associated with IgE reactivity leading to mast and basophil cell activation. This causes secretion of histamine, tryptase, and chymase which leads to a variety of physiologic responses.

Hypersensitivity reactions to banana have also been associated with birch pollen and latex allergies [5]. In some patients allergic to birch pollen, upon eating banana can develop itching and inflammation of the mouth and throat called oral allergy syndrome. The Mus a 1 gene in banana was found to be the most notable mediator for the IgE cross-reactions between the pollen and banana [5]. Nearly 20%–50% of people with natural rubber latex allergy have shown hypersensitivity after eating banana [5]. There has been evidence of cross-reacting allergens in latex and banana, but most incidents of banana allergy have been related proflin susceptibility with banana-latex association being less common [5]. Notably, our patient described symptoms of oral allergy syndrome and had positive IgE to latex and birch along with banana. Currently, there are no treatments or cure for banana allergy. Processing procedures such as steam boiling, microwave heating, enzyme treatment, and ethylene treatment help to reduce banana allergenicity, but need to be enhanced and further studied [5]. Current management is based on complete avoidance after the allergen has been identified.

We believe this to be the first case of Kounis syndrome due to banana allergy. Our patient underwent workup of her chest pain for over 1 year prior to allergy referral, testing and subsequent diagnosis. Removal of the offending agent resulted in cessation of her anginal symptoms. In Type II and III, early detection of Kounis syndrome could have significant impact on morbidity and mortality. This case highlights the importance of considering allergic causes of angina and allergy referral in an atopic patient with chest pain. It also emphasizes the importance of a complete history of present illness and to consider food allergy, especially banana, as a cause of Kounis syndrome.

## Data Availability

The data used to support the findings of this study are available from thecorresponding author upon request.
